# Supplementary material for: Analysis of the applicability and utility of a gamified didactics with exergames at primary schools: Qualitative findings from a natural experiment
Source: PLoS One. 2020 Apr 10;15(4):e0231269. doi: 10.1371/journal.pone.0231269 (PMC7147727; doi:10.1371/journal.pone.0231269)
Supplement: S1 Material — (DOCX) [file pone.0231269.s001.docx]

**Operational definitions of the teacher classification tree**

**1. Applicability**: refers to the ability of the gamified exergaming intervention to be carried out at school easily [facilitators] with minimum obstacles [barriers], with the greatest teacher predisposition [attitude, expectation of use and continuous learning], enduring in time [continuity] to develop the educational curriculum.

**1.1.** **Exergaming**: refers to the ability of the exergame to be carried out at school easily [facilitators], with minimum obstacles [barriers], with the greatest teacher predisposition [attitude, expectation of use and continuous learning], to develop the educational curriculum.

**1.1.1. Facilitators:** refer to any aspect that facilitates the application of the exergaming intervention at school.

**1.1.2. Barriers:** refer to any aspect that hinders the application of the exergaming intervention at school.

**1.1.2.1. Logistics:** refers to any aspect of the school’s material resources or facilities and physical spaces; i.e.: “there are not enough smartphones for all the students”.

**1.1.2.2. Students:** refer to the belief of teachers that exergaming is common to students by creating more predisposition, i.e.: “students are used to video games”.

**1.1.2.3. Technical problems:** refer to any technical problem regarding exergames, i.e.: “the mobile does not detect Wi-fi”.

**1.1.2.4. Specific teacher training:** refers to any negative aspect associated with the specific teacher training in exergaming, i.e.: “there are no manuals to apply exergames in PE”.

**1.1.2.5. Critical education.** It refers to any negative aspect associated with children using exergames as digital technology, and to any negative aspect associated with children using exergame as digital technology, i.e.: “digital technologies generate additions”.

**1.1.2.6. Teaching dependence on technology:** It refers to any negative aspect associated with teachers using exergame as digital technology, i.e.: "the natural essence of physical education is at of risk of being lost"

**1.1.3. Usage expectations:** refer to the teacher's consideration to apply the exergaming intervention at school in their professional future by either manifesting will/lack of will or manifesting possibility/impossibility of doing this; i.e.: "I have other preferences in my physical education programming to exergaming".

**1.2.** **Gamification:** refers to the ability of the gamified intervention to be carried out at school.

**1.3. General didactic design:** refers to the ability of the gamified exergaming intervention to be carried out at school, without specifically paying attention to its exergame or gamification component.

**1.3.1. Strengths:** refer to any aspect that makes the gamified exergaming intervention in school feasible exactly as it is designed, without changing anything; i.e.: "the intervention design promoted cooperation despite the leaderboard".

**1.3.2. Changes:** refer to any aspect that makes the gamified exergaming intervention in school feasible by introducing changes and improvements in its design; i.e.: "Children should be allowed to choose more exergame songs”.

**2. Utility**: refers to the ability of the gamified exergaming intervention to produce concrete and direct effects of psycho-educational interest both as a whole [as a general didactic design] and through some of its characteristics [gamification, exergame, dance, etc.].

**2.1. Exergaming:** refers to the ability of the exergaming intervention to produce direct concrete effects of psycho-educational interest.

**2.1.1 Benefits:** refer to the ability of the exergaming intervention to produce positive direct and concrete effects of psycho-educational interest. Specifically on:

**2.1.1.1. Learning**: refers to the ability of the exergaming intervention to produce positive concrete and direct effects on learning; i.e.: “Children learned without feeling that much shame from exergaming”.

**2.1.1.2. Health:** refers to the ability of the exergaming intervention to produce positive concrete and direct effects on health, i.e.: “Exergaming promotes overall health”.

**2.1.1.3. Performing alternative leisure:** refers to the ability of the exergaming intervention to produce positive direct and concrete effects on performing alternative leisure. i.e.: “They will have more active digital leisure because of exergaming”.

**2.1.1.4. Promoting physical exercise**: refers to the ability of the exergaming intervention to produce positive direct and concrete effects on promoting physical exercise, inside and outside the classroom, i.e.: “Exergaming is a good strategy to promote physical activity”.

**2.1.1.5. Motivation toward learning:** refers to the ability of the exergaming intervention to create commitment and enthusiasm toward learning, i.e.: “I perceived more attention in students when they were dancing with the exergame"

**2.1.2. Prejudices:** refer to the ability of the exergaming intervention to produce negative direct and concrete effects of psycho-educational interest. i.e.: “Watching a screen for too long is harmful”.

**2.2. Gamification:** refers to the ability of the gamified intervention to produce positive direct and concrete effects of psycho-educational interest.

**2.2.1. Benefits as a method:** refers to the ability of the gamified intervention to produce positive effects as an educational method.

**2.2.1.1. Motivation toward learning:** refers to creating commitment and enthusiasm toward learning, i.e.: “students became more involved in homework to get badges”

**2.2.1.2. Promoting physical exercise:** refers to creating commitment and enthusiasm toward physical exercise both inside and outside the classroom. i.e.: “Sedentary students will start exercising at home due to gamification”

**2.2.1.3. Promoting cooperation:** refers to creating commitment and enthusiasm toward teamwork and helping others, i.e.: “students worked more as a team to gain group points”

**2.2.1.4. Facilitates teacher evaluation:** refers to the greater ease of the teacher's task to assess learning, i.e.: “the gamification board provide the teacher with daily and weekly information"

**2.2.1.5. Acquiring learning:** refers to creating more and/or better learning in students; i.e.: “students learned the dance steps better with feedback from the screen”

**2.2.2. Prejudices:** refer to the ability of the gamified intervention to produce negative effects as an educational method.

**2.2.2.1. Too much competitiveness:** refers to the ability of the gamified intervention to produce an over-competitive attitude in students, i.e.: “gamification creates a very competitive class climate”

**2.2.2.2. Dependence on points:** refers to the ability of the gamified intervention to change students’ goal to points, rather than learning; i.e.: “If we take away points, students no longer dance"

**2.3. Utility of the general didactic design:** refers to the ability of the gamified exergaming intervention as a whole to produce direct concrete effects of psycho-educational interest, without differentiating gamification from the exergame.

**2.3.1. For teaching-learning processes:** refer to the ability of the gamified exergaming intervention as a whole to produce direct concrete effects on didactic processes.

**2.3.1.1. Promoting curricular values in students:** refers to the ability of the gamified exergaming intervention as a whole to produce direct concrete effects on promoting values that the current curriculum aims to instil, i.e.: “the intervention did not promote effort and mutual help in students”.

**2.3.1.2. Resolving conflicts:** refers to the ability of the gamified exergaming intervention as a whole to produce effects on students’ ability to resolve social conflicts. i.e.: “students more autonomously worked in groups and resolved conflicts quickly”

**2.3.1.3. Solving problems:** refers to the ability of the gamified exergaming intervention as a whole to produce effects on students’ ability to resolve learning problems, i.e.: “students looked for group solutions to create the choreography”

**2.3.2. For health:** refers to the ability of the gamified exergaming intervention as a whole to produce effects on health, i.e.: “thanks to the intervention, students’ physical condition improved”.

**2.3.3. Performing alternative leisure:** refers to the ability of the gamified exergaming intervention as a whole to promote more physically active digital leisure, i.e.: “students play exergames more than classic video games”.

**2.3.4. Promoting physical exercise:** refers to the creation of commitment and enthusiasm toward physical exercise both inside and outside the classroom, i.e.: “Sedentary students will start exercising at home due to intervention”

**2.3.5. For motivation:** refers to teachers’ motivating perception toward the intervention. i.e.: “the intervention was very motivating for students”.

**3. Miscellany:** refers to all the comments that did not meet the criteria to be included in any previous category.

**3.1. Researcher contributions:** refer to the comments made by researchers, such as questions or explanations during interviews, i.e.: “Is exergaming as equally important as traditional sports?”

**3.2. Irrelevant information for this study:** It refers to the comments made by participants that were irrelevant to the study objectives, i.e. “Yes, for example, the other day I played football...".

Table 1. Descriptive analysis of the interviews with teachers.

| Categories | N Files | % Files | N lines | % lines |
| --- | --- | --- | --- | --- |
| 1. Applicability | 7 | 100.00 | 501 | 44.18 |
| 1.1. Exergaming | 7 | 100.00 | 231 | 20.37 |
| 1.1.1. Facilitators | 5 | 71.43 | 46 | 4.06 |
| 1.1.2. Barriers | 7 | 100.00 | 185 | 16.31 |
| 1.1.2.1. Logistics | 4 | 57.00 | 27 | 2.38 |
| 1.1.2.2. Students | 0 | 0.00 | 0 | 0.00 |
| 1.1.2.3. Technical problems | 6 | 85.71 | 57 | 5.03 |
| 1.1.2.4. Specific teacher training | 4 | 57.14 | 62 | 5.47 |
| 1.1.2.5. Critical education | 4 | 57.14 | 25 | 2.20 |
| 1.1.2.6. Teaching dependence on technology | 2 | 28.57 | 14 | 1.23 |
| 1.1.3. Usage Expectations | 0 | 0.00 | 0 | 0.00 |
| 1.2. Gamification | 1 | 14.29 | 7 | 0.62 |
| 1.3. General didactic design | 7 | 100.00 | 263 | 23.19 |
| 1.3.1. Strengths | 7 | 100.00 | 117 | 10.32 |
| 1.3.2. Changes | 7 | 100.00 | 146 | 12.87 |
| 2. Utility | 7 | 100.00 | 557 | 49.12 |
| 2.1. Exergaming | 7 | 100.00 | 296 | 26.10 |
| 2.1.1. Benefits | 7 | 100.00 | 283 | 24.96 |
| 2.1.1.1. Learning | 5 | 71.43 | 58 | 5.11 |
| 2.1.1.2. Health | 2 | 28.57 | 4 | 0.35 |
| 2.1.1.3. Performing alternative leisure | 5 | 71.43 | 48 | 4.23 |
| 2.1.1.4. Promoting PE | 6 | 85.71 | 78 | 6.88 |
| 2.1.1.5. Motivation toward learning | 7 | 100.00 | 95 | 8.38 |
| 2.1.2. Prejudices | 3 | 42.86 | 13 | 1.15 |
| 2.2. Gamification | 7 | 100.00 | 155 | 13.67 |
| 2.2.1. Benefits as a method | 7 | 100.00 | 130 | 11.46 |
| 2.2.1.1. Motivation toward learning | 6 | 85.71 | 58 | 5.11 |
| 2.2.1.2. Promoting PE | 1 | 14.29 | 15 | 1.32 |
| 2.2.1.3. Promoting cooperation | 2 | 28.57 | 15 | 1.32 |
| 2.2.1.4. Facilitating teacher evaluation | 3 | 42.86 | 16 | 1.41 |
| 2.2.1.5. Acquiring learning | 2 | 28.57 | 26 | 2.29 |
| 2.2.2. Prejudices | 3 | 42.86 | 25 | 2.20 |
| 2.2.2.1. Over-competitiveness | 2 | 28.57 | 20 | 1.76 |
| 2.2.2.2. Dependence on points | 2 | 28.57 | 5 | 0.44 |
| 2.3. Utility of the general didactic design | 6 | 85.71 | 106 | 9.35 |
| 2.3.1. For teaching-learning processes | 5 | 71.43 | 84 | 7.41 |
| 2.3.1.1. Promoting curricular values in students | 3 | 42.86 | 30 | 2.65 |
| 2.3.1.2. Resolving conflicts | 3 | 42.86 | 15 | 1.32 |
| 2.3.1.3. Overcoming problems | 4 | 57.14 | 39 | 3.44 |
| 2.3.2. For health | 0 | 0.00 | 0 | 0.00 |
| 2.3.3. Performing alternative leisure | 2 | 28.57 | 13 | 1.15 |
| 2.3.4. Promoting PE | 0 | 0.00 | 0 | 0.00 |
| 2.3.5. For motivation | 2 | 28.57 | 9 | 0.79 |
| 3. Miscellany | 4 | 57.14 | 76 | 6.70 |
| 3.1. Researcher contributions | 4 | 57.14 | 24 | 2.12 |
| 3.2. Irrelevant information for this study | 3 | 42.86 | 52 | 4.59 |

“N File”= number of documents with that variable; “% File” = percentage of files in which that variable was encoded; “N line” = number of lines encoded in each variable; “% line” = percentage of the total number of lines in all the encoded files.

**Operational definitions of the students classification tree**

**1.** **Applicability:** refers to the ability of the gamified exergaming intervention to be carried out at school according to students.

**1.1. General design:** refers to the ability of the gamified exergaming intervention to be carried out at school without specifically paying attention to its exergame or gamification component.

**1.1.1. Changes:** refer to the aspects that can be changed in the intervention to make it more applicable, i.e.: “a mechanism can be added so that mobile phones do not turn off when held by hand”.

**1.1.2. Strengths:** refer to aspects that must be maintained in the intervention as they make it more applicable, i.e.: “I really liked it when we were able to score our own green point”

**2.** **Utility:** refers to the ability of the gamified exergaming intervention to produce direct concrete effects of psycho-educational interest, both as a whole [as a general didactic design] and through gamification.

**2.1. General didactic design:** refers to the ability of the gamified exergaming intervention as a whole to produce direct concrete effects of psycho-educational interest, without differentiating among gamification effects.

**2.1.1. Enjoyment:** refers to the ability of the gamified exergaming intervention as a whole to produce effects on pleasure while learning. i.e.: “I was spending my time very fast because I had fun while dancing”

**2.1.2.** **On learning**: refers to the ability of the gamified exergaming intervention as a whole to produce effects on learning, i.e.: “I've learned many dance styles that I didn't know”.

**2.1.3. Academic performance:** refers to the ability of the gamified exergaming intervention as a whole to produce effects on academic performance, i.e.: “I think the video game has helped me to achieve a better choreography”.

**2.1.4. Perceived physical effort:** refers to the ability of the gamified exergaming intervention as a whole to change students’ perceived physical effort, i.e.: “When I danced in front of the screen, I felt less tired”.

**2.1.5. Usage expectations:** refer to students' consideration to use or play exergames out of school in their future, i.e.: “I'm going to buy the video game to play at weekends”

**2.1.6. Promoting physical exercise:** refers to the ability of the exergaming intervention to produce positive direct and concrete effects on promoting physical exercise both inside and outside the classroom, i.e.: “after these classes, I like to exercise more”

**2.1.7. Resolving conflicts:** refers to the ability of the gamified exergaming intervention as a whole to produce effects on students’ ability to resolve social conflicts, i.e.: “we tried to resolve disagreements because they gave us team points”.

**2.2. Gamification:** refers to the ability of the gamified intervention to produce direct concrete effects of psycho-educational interest.

**2.2.1. Advantages:** refer to the ability of the gamified intervention to produce positive effects of psycho-educational interest.

**2.2.2. Disadvantages:** refer to the ability of the gamified intervention to produce negative effects of psycho-educational interest.

**3.** **Miscellany:** refers to all the comments that did not meet the criteria to be included in any previous category.

**3.1. Researcher contributions:** refer to the comments made by researchers, such as questions or explanations during interviews. i.e.: “Is exergaming as equally important as traditional sports?”

**3.2. Irrelevant information for this study:** refers to the comments made by participants that were irrelevant for the study objectives, i.e. “Yes, for example, the other day I played football...".

Table 2. Descriptive analysis of the focus groups and interviews of students by differentiating among groups.

| Classification tree | Control Group | | | | Experimental Group | | | |
| --- | --- | --- | --- | --- | --- | --- | --- | --- |
|  | N File | % File | N line | % line | N File | % File | N line | % line |
| 1. Applicability | 9 | 100.00 | 103 | 13.27 | 8 | 88.89 | 124 | 11.12 |
| 1.1. General design | 9 | 100.00 | 107 | 13.79 | 8 | 88.89 | 124 | 11.12 |
| 1.1.1. Changes | 7 | 77.78 | 64 | 8.25 | 8 | 88.89 | 73 | 6.55 |
| 1.1.2. Strengths | 9 | 100.00 | 43 | 5.54 | 7 | 77.78 | 51 | 4.57 |
| 2. Utility | 9 | 100.00 | 650 | 83.76 | 9 | 100.00 | 126 | 11.30 |
| 2.1. General design | 9 | 100.00 | 650 | 83.76 | 9 | 100.00 | 856 | 76.77 |
| 2.1.1. Enjoyment | 9 | 100.00 | 147 | 18.94 | 9 | 100.00 | 169 | 15.16 |
| 2.1.2. Learning | 9 | 100.00 | 195 | 25.13 | 9 | 100.00 | 195 | 17.49 |
| 2.1.3. Academic performance | 9 | 100.00 | 125 | 16.11 | 9 | 100.00 | 181 | 16.23 |
| 2.1.4. Perceived physical effort | 9 | 100.00 | 75 | 9.66 | 9 | 100.00 | 117 | 10.49 |
| 2.1.5. Usage expectations | 1 | 11.11 | 3 | 0.39 | 8 | 88.89 | 72 | 6.46 |
| 2.1.6. Promoting PE | 9 | 100.00 | 61 | 7.86 | 8 | 88.89 | 82 | 7.35 |
| 2.1.7. Resolving conflicts | 9 | 100.00 | 44 | 5.67 | 8 | 88.89 | 40 | 3.59 |
| 2.2. Gamification | 0 | 0.00 | 0 | 0.00 | 7 | 77.78 | 99 | 8.88 |
| 2.2.1. Advantages | 0 | 0.00 | 0 | 0.00 | 7 | 77.78 | 93 | 8.34 |
| 2.2.2. Disadvantages | 0 | 0.00 | 0 | 0.00 | 2 | 22.22 | 6 | 0.54 |
| 3. Miscellany | 5 | 55.56 | 19 | 2.45 | 7 | 77.78 | 36 | 3.23 |
| 3.1. Researcher contributions | 5 | 55.56 | 12 | 1.55 | 7 | 77.78 | 20 | 1.79 |
| 3.2. Irrelevant information for this study | 1 | 11.11 | 7 | 0.90 | 3 | 33.33 | 16 | 1.43 |

“N File”= number of documents with that variable; “% File” = percentage of files in which that variable was encoded; “N line” = number of lines encoded in each variable; “% line” = percentage of the total number of lines in all the encoded files.
