# Supplementary material for: Analysis of the applicability and utility of a gamified didactics with exergames at primary schools: Qualitative findings from a natural experiment
Source: PLoS One. 2020 Apr 10;15(4):e0231269. doi: 10.1371/journal.pone.0231269 (PMC7147727; doi:10.1371/journal.pone.0231269)
Supplement: S2 Material — (DOCX) [file pone.0231269.s002.docx]

**Transcripts TEACHERS**

[The extracts used in the main article are marked in blue]

**Category: 1.1.1. Facilitators**

<Files\\01 Entrevista profesorado - D> - § 3 Coded references [Coverage 4,36%]

Reference 1 - Coverage 1,54%

¶5: We have a block and some objectives, and the exergame helped to work by facilitating a context, because you had quite a specific context and a place where you knew what you were going to do.

<Files\\11 Entrevista profesorado - J> - § 1 Coded references [Coverage 4,72%]

Reference 1 - Coverage 4,72%

¶10: Yes, I have already mentioned the possibility of obtaining second-hand mobiles. Obviously they are more economical. When I suggest obtaining second-hand [smartphones], I mean they can be borrowed from people, or donated. […]. So donations can be a possibility. It would just be a matter of organizing a small campaign with teachers, parents

… but we will see. I can’t see any disadvantage of doing this.

<Files\\18 Entrevista profesorado L> - § 2 Coded references [Coverage 5,02%]

Reference 2 - Coverage 0,69%

¶19: If I had the materials, yes. I really enjoyed it, honestly.

**Category: 1.1.2.1. Logistics**

<Files\\06 Entrevista profesorado S> - § 2 Coded references [Coverage 3,61%]

Reference 1 - Coverage 1,66%

¶5: Of course, the limitation of having to rely on mobile phones; the limitation of the material, I believe that we at schools are not ready for all this, as we do not even have proper networks”, nor are there properly equipped places.

**Category: 1.1.2.3. Technical problems**

<Files\\01 Entrevista profesorado - D> - § 5 Coded references [Coverage 9,48%]

Reference 1 - Coverage 0,72%

¶11: I think that its main problem is that it involves a number of minimum technical elements.

<Files\\11 Entrevista profesorado - J> - § 1 Coded references [Coverage 5,06%]

Reference 1 - Coverage 5,06%

¶8: Negative aspects; it is obviously hard to set up. I don’t agree with them having mobile phones and being able to play with their phones, not in year 5, year 6, nor even in Secondary Education, especially in years 1 and 2. So if I had to set up the unit, I´d have trouble obtaining enough mobiles so they can do this autonomously, and I could do this as a teacher. Perhaps the school can offer this service.

**Category: 1.1.2.4. Specific teacher training**

<Files\\06 Entrevista profesorado S> - § 3 Coded references [Coverage 9,41%]

Reference 1 - Coverage 3,82%

¶15: I will try it, but my knowledge of technology is limited. So I would use it if I was sure about the content I´m teaching. I find it interesting, but you must be quite clear about this. If I was clear about what I wanted to achieve and how to go about it, of course I would use it, but only if I could clearly see it. No because “today we´re doing a session with mobiles; cool; we are going to play with mobile phones and we have them”. I honestly think we would be losing the educational side.

Reference 2 - Coverage 2,24%

¶16: You see in my case, I don’t control the themes, it’s not a content I handle. I have never played and I don’t know how to play. So my knowledge is very poor. Some children have plenty of knowledge about video games. If I know how to provide the educational approach, these students could be brilliant helpers.

Reference 3 - Coverage 3,34%

¶22: It is true that when you presented the project to us, at first I thought it would be impossible because I did not understand it. I mean, I did not understand its dynamics, how it could be organized. Then I had doubts at first about how it would work, how it would end up. All this with technological limitations, for our school, the conditions it offers, the student, installation and group characteristics, the dynamics among teachers, etc.

<Files\\11 Entrevista profesorado - J> - § 1 Coded references [Coverage 8,13%]

Reference 1 - Coverage 8,13%

¶24: It is all worthwhile because it may all be very interesting. But then you have to choose, give quality, and time is limited. Motivation is also limited because now there are many open fronts with new methodologies. Remember what we have talked about in relation to thought routines or cooperative work; we have to also introduce cooperative work. We are introducing units based on cooperative work techniques. There are so many things. So right, yes, it can be introduced, but it’s not easy. It is not a priority right now. What is more a priority is how to introduce a unit of dances with what that entails, and exergames are another support than the actual exergames themselves. Everything is worthwhile and perfectly valid, but we have to prioritize.

<Files\\12 Entrevista profesorado - J> - § 2 Coded references [Coverage 15,99%]

Reference 2 - Coverage 5,82%

¶27: I don’t think it’s the most immediate priority. Right, I´m looking it from our school’s training point of view. Of course, they don’t have to be other school’s points of view. Many teachers had B2 and now have C1. As the school is bilingual, well we prioritized this. Now that priority no longer exists. We have been training in comprehension projects. We have trained in cooperative work. In fact you´ll have noticed that all classrooms are cooperative, and each student may play a different role. So it depends on the centre’s priority lines. I don’t think that exergames is a priority line for the PE department, simply for priority reasons, and because PE can be done well, and it can be made motivating and creative without having to work with exergames. I don’t believe it is an immediate priority.

**Category: 1.1.2.5. Critical education.**

<Files\\12 Entrevista profesorado - J> - § 2 Coded references [Coverage 6,71%]

Reference 1 - Coverage 4,09%

¶5: Well positive because, besides, I believe that video games are not well considered socially speaking. I think that people believe video games create addictions, entail problems and what have you. So how to give a point of view that also favors healthy lifestyles. We are talking about dancing, performing physical activity, sport. So I think that, let’s say, the point of view it offers changes it a bit, don’t you? It is like combining two things they like, changing the negative parameter, let’s say the game. Because, at the end of the day, they use it to practice physical activity. Which is exactly the opposite to what other video games achieve.

<Files\\17 Entrevista profesorado N> - § 1 Coded references [Coverage 2,61%]

Reference 1 - Coverage 2,61%

¶21: Yes. Let’s say opting for an activity that was… Yes. Perhaps we adults spot a difference between sport and video games. And if we did not find this difference, they might play something else. Perhaps they would accept if we offered it to them.

**Category: 1.1.2.6. Teaching dependence on technology**

<Files\\06 Entrevista profesorado S> - § 1 Coded references [Coverage 3,19%]

Reference 1 - Coverage 3,19%

¶10: Disadvantages, apart from practical ones, include us depending on technology that is, therefore, a resource we cannot control. When you are teaching, you manage your class, you manage times. As soon as you depend on networks, spaces and a technology, times differ. So it’s true that some sessions were small, or perhaps making good use of sessions is sometimes not so …

<Files\\07 Entrevista profesorado J> - § 2 Coded references [Coverage 13,65%]

Reference 2 - Coverage 9,05%

¶7: So I feel that such learning is not rich in this sense. I prefer nature myself, playing with sticks, stones, etc., which we are missing out on. With the few hours we spend on PE, I think we should focus on forgetting to use mobile phones, technologies, and we should act as a group that only interacts”. Because I believe that, right now, there is no subject in the curricula in Aragón that is about this.

**Category: 1.3.1. Strengths**

<Files\\01 Entrevista profesorado - D> - § 4 Coded references [Coverage 13,23%]

Reference 2 - Coverage 2,31%

¶11: However, everything can be solved. It can be done in groups with everyone dancing, and this week we have this song; then on such and such a day, you haven’t got exergame, so you have the time to practice and it’s your turn first next week, and you have to make so much effort. I mean, there are different ways of using it, but it involves that planning.

Reference 4 - Coverage 4,07%

¶15: In the end, exergame is a gamification process, a methodology that you can consider in one way or another. You can maintain Just Dance, and stars can be given like a co-evaluation. Or other groups can hand out stars. For instance, they can be two gommets and a decision is made to give them according to the goal. But considering gamification and using technologies to develop units should not be a problem, not for corporal expression as we have just done, but dancing.

<Files\\06 Entrevista profesorado S> - § 4 Coded references [Coverage 10,11%]

Reference 3 - Coverage 4,30%

¶16: But I think it is interesting. In many of these contents, students, and there are many more Secondary Education students, but also Primary Education students, students from higher cycles, can be good helpers because they are technologically better trained than we are. So perhaps some of these students acting as helpers could be a resource because they have certain knowledge. Then you need previous preparation work, knowing well what we want to do, how we want to do it, students who are more used to dealing with exergame, video games…, students can perhaps help you during this session.

<Files\\07 Entrevista profesorado J> - § 2 Coded references [Coverage 15,02%]

Reference 1 - Coverage 6,39%

¶3: Well. I liked it, especially children’s reactions when working in PE with a means like technology. You can really see that they live in the technology era, and their interest and motivation quickly grow when using this small*...* … So, in short, I liked it.

<Files\\12 Entrevista profesorado - J> - § 2 Coded references [Coverage 15,50%]

Reference 1 - Coverage 5,14%

¶17: Yes, I believe that virtue lies in mixing things. Not just one thing, not all exergames. ... precisely what we have done. It might be better doing half a unit as always and then adding strokes of exergames, why not? Or alternating. I mean, the way the unit is contemplated already differs. But I don’t see why we can’t mix both things. Yes, we could do that no problem. Moreover given the experience, which has been positive, we have the space and resources to use it, so there would be no problem. Now we have this, and we have performed it in the Assembly Room, we can change and try it somewhere else. Or we can move it. But then again, if you don’t try doing that, you can’t see the failures or benefits.

<Files\\17 Entrevista profesorado N> - § 4 Coded references [Coverage 16,71%]

Reference 1 - Coverage 2,34%

¶3: I quite liked it because we began with simple things that were related. I mean nothing isolated was included, nothing that did not work toward meeting the ultimate objective, rather everything worked toward that objective.

Reference 2 - Coverage 4,80%

¶25: I don’t think that the students from years 5 or 6 are better prepared. I think the year-6 students wanted more and were more autonomous and you did not need to tell them off or be on top of them as much by saying «this must be done, that must be done». The year-5 students did not find it so easy. Perhaps it was because the four groups were in the same classroom and the year-6 students were divided into two, which is easier to control. But being prepared, well I think that both have had enough resources to master the dance.

<Files\\18 Entrevista profesorado L> - § 3 Coded references [Coverage 6,18%]

Reference 1 - Coverage 2,13%

¶3: My first impressions were positive because I noticed that students were really motivated; I noticed a sequence of activities; I also noticed that they never lost the gist.

Reference 3 - Coverage 0,73%

¶17: But there is also another type of activities. So, of course.

**Category: 1.3.2. Changes**

<Files\\07 Entrevista profesorado J> - § 1 Coded references [Coverage 21,41%]

Reference 1 - Coverage 21,41%

¶17: No. Perhaps, I´m not sure if it is our problem or the school’s problem but perhaps before beginning the unit, who is in charge of managing spaces, this must be made quite clear, right? Good, you are in charge of this. And then this person is in charge of managing spaces throughout the unit. Because I found that this was a problem. Of course there is very little time at the end, and with technology included, you have to expect to switch on all the devices and that, so I think we could have better gained some more time for these management aspects. I mean “not here now, not there now, not…”, in these minor matters. Particularly to set limits. It’s quite easy. For example, assign one teacher. If you tell me at the very start of the unit you do not have to be in charge of it, it´s one task less. It might even be easier for one teacher working at the school, someone who knows where to go or which teachers, and it’s one task less for you to do. One person for planning and they say in this planning that…

<Files\\12 Entrevista profesorado - J> - § 3 Coded references [Coverage 15,48%]

Reference 3 - Coverage 8,21%

¶23: I can only think that we have had a blunder here that had to be overcome. For example, some events at schools are unavoidable, like the International Day of Peace, which they told us about yesterday as we went out during playtime. So classes start 30 minutes later. By simply having these things, if possible, because sometimes it’s not possible as the school’s life is the school’s life, but if possible, we can control such events. Precisely because of what we were talking about before, for the required logistics, organization, especially for materials and spaces. As for the rest, I think it has been done well. It all worked well for us and, fortunately, the means you have when you have brought mobiles and the rest; the spaces we have used; projectors; Wi-fi… So it has probably gone well. It’s just that when it is necessary to prepare things for PE, I always arrive much earlier, and I try to leave the gym ready before a class precisely to play with that motivation. It’s not the same as entering and having to set things up, than entering and saying “wow”. You see that, especially with the young ones and the circuit. When they see everything set up, it´s a plus. So I would go further as to whether you can calibrate in centers, which I know is difficult, or even impossible, in many aspects.

**Category: 2.1.1.1. On learning**

<Files\\01 Entrevista profesorado - D> - § 6 Coded references [Coverage 9,50%]

Reference 1 - Coverage 0,89%

¶5: No, for me exergame is an instrument you can use to fulfill the objectives you set out, your educational purposes.

Reference 2 - Coverage 0,39%

¶5: are technical elements to develop the curriculum.

<Files\\11 Entrevista profesorado - J> - § 1 Coded references [Coverage 1,21%]

Reference 1 - Coverage 1,21%

¶8: We have not said this before, but I have worked my coordination very well by dancing in parallel to the screen.

<Files\\17 Entrevista profesorado N> - § 2 Coded references [Coverage 8,22%]

Reference 1 - Coverage 7,81%

¶5: Then you see different dance steps and they no longer feel so ashamed because they are not being watched while their classmates dance, rather everyone is watching the screen and they feel more capable because they are immediately recognized by scoring points. However with traditional dancing, you see they do not master it as well and do not feel “I´m doing it perfectly”. Depending on which group it is, they may feel more or less skilful. Unlike using a mobile because it doesn’t matter what your classmates are like as you’ve got your points. That’s your reference of how you do it. Then the game’s dance steps were included. And that’s another benefit because the Class Dojo points also motivate a lot, but I can’t think of any more. As for them dancing more or less to the rhythm, I think they were similar.

Reference 2 - Coverage 0,41%

¶31: Oh, yes. I would use exergames as a resource.

**Category: 2.1.1.3. In performing alternative leisure**

<Files\\07 Entrevista profesorado J> - § 1 Coded references [Coverage 9,82%]

Reference 1 - Coverage 9,82%

¶14: Yes, but it’s like what I said before. I think it’s another form of physical activity, which is very interesting for those boys and girls who have no other way to practice physical activity. If there was some way of performing physical activity in the natural environment, with other classmates, don’t let technology be involved, then I’d firmly go for that option. However for those students who spend many hours at home, it’s an excellent way for them to spend their free time.

<Files\\18 Entrevista profesorado L> - § 2 Coded references [Coverage 19,05%]

Reference 1 - Coverage 13,20%

¶9: So, off the top of my head, I think that many of the activities we do, we have to deal with them, don’t we? I mean, they have to be done in class, in which case, I think that if we talk to them and get them to instill this movement routine, they can do it at home. Of course this can be applied to many other types of games too. But, unfortunately, it would appear that children go outdoors less and we get them to move … I personally believe that if they go outdoors more to perform different physical activities in the natural environment, so much the better. But if we give them an alternative at home that they can move with, they can develop this physical activity, because we are giving them an opportunity, a chance to move. Therefore, it’s positive. It’s simply an alternative. I´m not comparing, no, not at all. But I personally think that it is much better to perform physical activity outdoors, but I believe that your work is an alternative for those kids who, for whatever reason, do not spend much time outdoors. They can do this autonomously as this type of units can be a form of learning so they can develop it on their own. When I say outdoors, I generally mean the physical activity performed outside an enclosed area.

Reference 2 - Coverage 5,84%

¶29: I hope not, I hope not, but it’s possible. I´m not sure about how to get involved in this matter. It’s possible. Yes, it is, but not for me. Well, if it involves movement and it keeps us entertained and it’s fun, then OK. But I find it hard to admit that, that’s how I feel. I´d find it hard to admit saying Yes because it is another tool and a very important window, but I think that we have plenty of very interesting resources apart from technology to make movements and to perform physical activity. I think it’s not necessary, but an option. I´m not sure if I´m making myself clear.

**Category: 2.1.1.4. On promoting physical exercise**

<Files\\18 Entrevista profesorado L> - § 3 Coded references [Coverage 13,67%]

Reference 2 - Coverage 4,33%

¶17: I think that any advantages that we embrace for students’ knowledge are positive. And it might be more representative for some students than for others. So, is this any different? Yes. Is it considered a form of physical activity? Yes. So my answer is yes. I think that offering new windows to learning is positive. I´d propose anything that matches our contents and objectives.

Reference 3 - Coverage 2,53%

¶33: It is true that no-one sat down with Just Dance Now. I don’t like admitting this, because I defend people as a being… but when we danced, some sat down and I had to make some of them get up off the floor and ask «what are you doing?»

**Category: 2.1.1.5. Motivation towards learning**

<Files\\01 Entrevista profesorado - D> - § 2 Coded references [Coverage 12,68%]

Reference 1 - Coverage 12,52%

¶7: Well on the one hand, there is the novel part, right? Of course, you have a new motivating instrument, and you can see on the screen bright images; then people are dancing, models, because you see boys and girls, so to speak, who dance well, even though you can’t identify figures. We are talking about the figures in Just Dance, and you’d see girls and boys dancing very well, and also lots of fun choreographies. So it would be like a model to follow, because this is a guided class. We’re not talking about there being any methodological innovation, because this is the aerobics we´ve always known. The thing is, the model you have is firstly very visually striking because sceneries change, who dances changes, it’s very entertaining, it gives points… These things are highly motivating and they certainly encourage children to connect to an activity. Having a mobile in their hands allows them to follow the dance, it automatically tells them what they have achieved, and they obtain immediate results. This means that the boy or girl consider an improvement measure or a measure of the quality of their efforts and their learning. I mean, it provides them with feedback, which is immediate. Especially with what I see when having to work, which is motivating. When using an element, but not just having a phone in my hand, but also what is visual. Then there is the feedback given when scoring: you finish and you have points. Then you think, well that’s it, and you know if you have done it well, or badly. Then you think I´m going to dance again and see if I can get a better score … I think that this feedback is very important too.

<Files\\06 Entrevista profesorado S> - § 1 Coded references [Coverage 3,47%]

Reference 1 - Coverage 3,47%

¶9: Well, with benefits firstly in motivation because our students get their hooks into anything technological. They’re used to working with new technologies. So perhaps it is not the material we have or normally use in our PE area, which is something in its favor, especially when students’ predisposition is better. So, OK, it’s positive in that sense. We have incorporated technologies that are being used in other classes.

<Files\\18 Entrevista profesorado L> - § 4 Coded references [Coverage 20,00%]

Reference 1 - Coverage 7,78%

¶7: Yes, I personally want to do similar things if I can. This means that I believe in relying on technology for PE. We´ll see what conclusions you reach but, off the top of my head, I think it’s very beneficial. Especially when starting because kids find it appealing. Perhaps this is very difficult nowadays as they feel 100% stimulated and seem to already know everything, despite the fact that they like a simpler game the best later on. Complexity does not need to be the most interesting point. But, in principle, I have seen that linking technology and education, PE in this case, is possible. Yes, it’s possible and it is an interesting background to work on.

**Category: 2.1.2. Prejudices**

<Files\\07 Entrevista profesorado J> - § 1 Coded references [Coverage 4,52%]

Reference 1 - Coverage 4,52%

¶5: Let’s see. My view about technologies and video games is very radical. So in line with my view, its benefit is that you have motivation and interest. I don’t know if there are more benefits.

**Category: 2.2.1.1. Motivation towards learning**

<Files\\06 Entrevista profesorado S> - § 4 Coded references [Coverage 8,65%]

Reference 2 - Coverage 1,63%

¶13: I thought that the gamified system was good because first, what is prioritized is that you improve. You have a baseline level and you obtain an end level. You have some results. You see what progress you make.

<Files\\11 Entrevista profesorado - J> - § 2 Coded references [Coverage 3,12%]

Reference 1 - Coverage 0,76%

¶12: Yes. Yes, in general, the points system is motivating. It’s motivating.

**Category: 2.3.1.3. To solve problems**

<Files\\11 Entrevista profesorado - J> - § 2 Coded references [Coverage 14,94%]

Reference 1 - Coverage 8,72%

¶6: Then lack of inhibition comes. The truth is it has given them lack of inhibition, and perhaps this would prove more difficult with another type of unit. In Secondary Education, the first course, I go to Secondary Education because it’s where I work more on dances. What they do in both years 1 and 3 is watch a video. I don’t teach them to dance, they learn from watching a video. Then there’s the structure. I give them a script to structure it, the steps there are, the dance formations there are… and they learn watching a video. But there always comes a point when they have to end up learning to dance by moving about. That is the point they find hard. They find this lack of inhibition part hard. Then they later have to teach others. That is where the barrier appears, which they find a bit hard to overcome. With exergame, this shame barrier is quickly overcome.

**Transcripts STUDENTS CONTROL GROUP**

**Category: 1.1.2. Strengths**

<Files\\20 Grupo discusión alumnado 5º 2> - § 2 Coded references [Coverage 6,96%]

Reference 1 - Coverage 5,05%

¶6: D. I personally liked it a lot because I had never done anything like this. We have always done sport games in playtime, and this year has been the first time we have used dance. It has been great fun.

**Category: 2.1.1. On enjoyment**

<Files\\14 Grupo discusión alumnado - 5º> - § 2 Coded references [Coverage 34,36%]

Reference 1 - Coverage 26,69%

¶3: A. I thought they were great fun, but less the group one because they fought a lot.

¶4: D. I agree with her.

¶5: A. They fought a lot. They couldn’t care less.

¶6: D. It was the same in my group.

¶7: A. I liked it, if it was not for this problem.

¶8: D. I didn’t like being with (x). He’s too pushy.

¶9: B. I liked it. I liked it very much. The whole thing. The only thing is they held your hands too tightly to dance.

¶10: D. She likes whatever dances they put on.

¶11: B. Yes, we both like them because we have been to dances.

¶12: D. I like fooling around and dancing.

¶13: C. I liked them all. For me, the best was the group part because I was paired with a very good group. It was a pity that some did not do it well. The only one I didn’t like was “to the right”, because you had to move a lot.

¶14: D. I liked all the corporal expression ones, except the first one, the one you had your eyes blindfolded and did what you wanted. I felt a bit ashamed, even though no-one was watching. I felt ashamed just thinking about it. I didn’t feel at ease. It was a bit like dancing in front of an audience and, although my eyes are blindfolded, I did not feel at ease. It was not like being alone, as if they could actually see me.

¶15: C. Yes, you felt it.

¶16: D. In the end I did. I´m happy messing about while I dance. At times I did festivals in Barcelona, because I´m Catalan and live in Barcelona, and I was ashamed when I did do some dance steps badly and colleagues laughed. What I liked the most was salsa, and learning such as complicated quick dance.

<Files\\20 Grupo discusión alumnado 5º 2> - § 3 Coded references [Coverage 19,29%]

Reference 1 - Coverage 17,26%

¶3: A. Let’s see. I really enjoyed dancing salsa because we were really making fools of ourselves, but also having fun. Then we also danced with partners, which was great fun.

¶4: B. Yes, it was fun.

¶5: C. I really liked salsa because I was having fun with my friends and because it is a dance we´d never practiced before. We didn’t get it quite right and we were laughing about ourselves and them.

¶6: D. I really liked it because we have never done anything like this before. We had always done sport games in playtime, but this year is the first time we have done dances. It´s was so much fun.

¶7: C. I liked it because we were all together.

**Category: 2.1.2. On learning**

<Files\\14 Grupo discusión alumnado - 5º> - § 4 Coded references [Coverage 17,48%]

Reference 3 - Coverage 3,82%

¶20: A. I felt ashamed. I mean, before I focused more on what people would think, but not anymore. I don’t really mind what they think now.

¶21: B. I feel the same. Before I wondered what people would say about me, but I don’t do that so much now.

**Transcripts STUDENTS EXPERIMENTAL GROUP**

**Category: 1.1.1. Changes**

<Files\\05 Grupo discusión alumnado> - § 3 Coded references [Coverage 6,89%]

Reference 3 - Coverage 2,09%

¶48: C. What I didn’t like was having to hold my phone all the time, and it switched off.

<Files\\10 Grupo discusión alumnado 5º> - § 2 Coded references [Coverage 8,79%]

Reference 1 - Coverage 4,45%

¶3: And then what I did not like much was that while others were dancing with mobile phones, some others had to dance with no phone. This was no good because you didn’t score points.

**Category: 1.1.2. Strengths**

<Files\\05 Grupo discusión alumnado> - § 3 Coded references [Coverage 3,68%]

Reference 1 - Coverage 0,78%

¶5: B. I generally liked everything.

<Files\\16 Grupo discusión alumnado 6º grupo 02> - § 3 Coded references [Coverage 11,90%]

Reference 1 - Coverage 4,89%

¶32: D. Just Dance Now helped us to learn new dance steps.

¶33: A. It has taught us new steps to apply them in the dances we do. And being on stage too.

Reference 3 - Coverage 1,92%

¶67: B. It helps you see how you’ve improved, and that. So much the better if we do it badly.

<Files\\23 Grupo discusión alumnado 6º 1> - § 5 Coded references [Coverage 7,31%]

Reference 2 - Coverage 2,51%

¶26: C. Of course, but now it’s different. We like it because it is something we’ve never done before. We’ve never tried doing things like this, dancing in PE.

¶27: D. and with video games.

¶28: C. Then they gave you points, which I liked a lot.

Reference 5 - Coverage 2,39%

¶134: C. I quite liked it. I also liked the idea that an application is used in which we can see for ourselves if we want to see our points, rather than having to ask about them. I didn’t look, but I quite liked it.

<Files\\25 Grupo discusión alumnado 6º 3> - § 3 Coded references [Coverage 10,14%]

Reference 3 - Coverage 6,60%

¶71: D. I think it’s great, and you feel more motivated when they give you an individual point.

¶72: B. I agree and think that you are really motivated to do it better, to try to do it well. And, of course, you do.

¶73: D. You feel motivated before being given a point. Apart from feeling motivated to win points, you must also motivate your team so that it gets plenty of points. And team members win. For example in my team, which is green, there were two colleagues who got lots of points and that. I think (x) has came came first in the class.

¶74: C. I liked it because you felt motivated to work better, especially for double points. I made the same effort, but felt more motivated to do it well.

**Category: 2.1.1. On enjoyment**

<Files\\03 Entrevista alumnado - Á> - § 3 Coded references [Coverage 16,53%]

Reference 3 - Coverage 4,34%

¶16: I like playing Just Dance Now more because it’s like a video game. I´m used to playing video games, and I more or less know how it works.

<Files\\15 Grupo discusión alumnado 6º grupo 01> - § 1 Coded references [Coverage 11,39%]

Reference 1 - Coverage 11,39%

¶3: E. Well, I found it a bit hard because when it was your turn to perform corporal expression, you think «I don’t know what to do.

¶4: C. I found dancing easier.

¶5: A. It’s been great fun, at least I thought so.

¶6: D. I think that dancing was fun because we got ideas from Just Dance Now and that.

¶7: B. I also thought it was great fun. We didn’t do this at my last school.

<Files\\24 Grupo discusión alumnado 6º 2> - § 3 Coded references [Coverage 22,13%]

Reference 1 - Coverage 12,98%

¶3: D. I thought it was great and really fun because you play as if it was a video game at school, moving about, it was cool.

¶4: A. It’s been really cool because, apart from enjoying dancing a lot, and a lot of people like it […], you are also practicing sport for your health. And I think that’s great.

¶5: E. I quite enjoyed it because, apart from it being a video game, you can quite enjoy yourself, you can learn to dance.

¶6: C. I thought it was good and practical also for children who don’t know how to dance, and they can learn a bit about one part, and know more about another. For example, in Just Dance Now, if you don’t know how to dance and you don’t know the movements to create a song. I mean with a dance, like we have been doing. Then you have ideas about what you can do next.

¶7: B. I really enjoyed it because you can express yourself with your body and transmit sadness, rage.

¶8: D. It has also taught us, for example, when to smile, musical rhythms, sentences, how to end a song, how to start one …

¶9: A. Yes, because if a song is, for example, sad, you have to express it by dancing.

¶10: C. Yes, it’s cool.

¶11: A. You are happy, smiling, and enjoying yourself.

¶12: C. If it has rhythm, it’s rock music for example, then movement speeds up, like that.

**Category: 2.1.2. On learning**

<Files\\05 Grupo discusión alumnado> - § 3 Coded references [Coverage 31,78%]

Reference 1 - Coverage 9,79%

¶20: D. No, because I didn’t feel ashamed.

¶21: C. A bit. But it was OK at the end.

¶22: B. Sometimes, I mean before I felt ashamed to dance with people around me. But when we worked with Just Dance Now, I no longer felt ashamed because I danced casually.

¶23: A. I was so ashamed before, but not now. Before I was afraid of listening to songs of terror at night, but I don’t mind listening to them now.

**Category: 2.1.3. Academic performance**

<Files\\03 Entrevista alumnado - Á> - § 2 Coded references [Coverage 14,38%]

Reference 1 - Coverage 9,07%

¶7: l like dancing because I liked dancing before. But I didn’t like corporal expression much, that bit with zombies. I think my dancing has improved, and I have learned new steps. I have improved a bit in PE in general because we move differently, and we can move parts of your body in another ways.

<Files\\05 Grupo discusión alumnado> - § 3 Coded references [Coverage 6,22%]

Reference 3 - Coverage 2,90%

¶45: A. My arm ached holding the phone. But sometimes when dancing, yes, because I got quite involved. And then in PE…

<Files\\16 Grupo discusión alumnado 6º grupo 02> - § 3 Coded references [Coverage 28,95%]

Reference 2 - Coverage 10,08%

¶11: C. Yes.

¶12: D. No, because I liked it the same

¶13: E. I now like [dancing] much more. Before, I didn’t feel like letting myself go. But when we started dancing with Just Dance Now, I had the chance to …

¶14: A. You express yourself.

¶15: E. Yes, to express myself.

¶16: C. I liked it more because I have never danced, but I would now.

¶17: A. I liked it more. Before I did not wish to express myself, but I do now.

**Category: 2.1.4. Perceived physical effort**

<Files\\24 Grupo discusión alumnado 6º 2> - § 1 Coded references [Coverage 13,56%]

Reference 1 - Coverage 13,56%

¶56: E. Well, some…

¶57: D. It depends on the dance because some were tiring.

¶58: C. For instance, Just Dance, the last ones made me feel more tired, obviously because they’re a bit like on a higher level.

¶59: A. You started slowly and then worked your way up.

¶60: D. I remember when we danced that “break free” one when we have to move our arm a lot to get points.

¶61: C. Oh the “That Power” one.

¶62: B. I found that the sessions weren’t that tiring.

¶63: A. I prefer those of Just Dance than those of the other one.

¶64: E. Yes, so do I.

¶65: D. I ended up sweating with both, but I felt more tired with Just Dance.

¶66: C. I can’t decide between one and the other because they all seemed more or less the same to me. But one involves doing a bit more, I mean, making more effort, and the other a bit less. But I thought both were nearly the same.

¶67: B. I felt more tired in Just Dance.

¶68: A. Well, in dancing too.

¶69: B. Well if you repeated the dancing a lot and spent a long time on it, you’d be tired. But if you say, “come on, let’s try introducing this or the other”, then as you would be looking, concentrating, you’d not use so much energy.

¶70: E. But you did not think about feeling tired with Just Dance Now.

¶71: D. I remember that my group could not decide during the first sessions, and we were loudly discussing “I don’t know, this one, or that one”. Then we decided and…

¶72: A. Yes, because we didn’t even have the initial position on the first day.

<Files\\26 Grupo discusión alumnado 6º 4> - § 1 Coded references [Coverage 23,52%]

Reference 1 - Coverage 23,52%

¶41: D. No.

¶42: C. No.

¶43: B. No.

¶44: A. No.

¶45: D. Let’s see, they were slower.

¶46: C. They were normal, some were good.

¶47: B. There was a mixture.

¶48: A. Some were stronger, that’s true.

¶49: C. It also depends on how much strength you use.

¶50: A. Some classes were more tiring. If you made more effort, then…

¶51: D. Yes, some people felt tired earlier.

¶52: B. I think I felt more tired in the wooden hall.

¶53: C. The video game tired me more.

¶54: D. The same happened to me.

¶55: B. I felt more tired in the hall because it was constant repetition. In Just Dance, you repeat once, but then change, but not with the other, where you always do the same thing.

¶56: C. I [felt more tired] with Just Dance Now because you have to concentrate more on the game and you had to do everything you saw on the screen. That’s more overwhelming. So of course you felt more tired.

¶57: D. Because in the other one, it was about how I invented dance steps. At the end, they were all repeated, and you felt calmer.

¶58: C. You have more freedom to work.

¶59: D. If you forget something, it doesn’t matter. But as Just Dance gives you points, you lose points if you forget a part or stop moving your hand.

**Category: 2.1.5. Usage expectations**

<Files\\03 Entrevista alumnado - Á> - § 2 Coded references [Coverage 25,58%]

Reference 1 - Coverage 16,38%

¶15: Yes. I played it the second week after we did in class. I have started playing Just Dance Now at home. I didn’t know it before. I didn’t know this application and I like it. At home, I sometimes play with my cousin, and alone on other occasions. Now we’re in week 3 or 4. Then we play together and need more devices like mobiles to play with more people. We dance in the living room. We meet to go to school, and she meets me when we come from datchball and then we play. We play with the TV, the PC and my mobile, and with my mum’s mobile.

<Files\\23 Grupo discusión alumnado 6º 1> - § 1 Coded references [Coverage 11,55%]

Reference 1 - Coverage 11,55%

¶142: C. I think that both things are good. If I had to choose, I prefer to go outside because, in the end I´m with them and I move about.

¶143: B. And you enjoy yourself.

¶144: C. You have fun, of course. You don’t relate with others in the video game.

¶145: D. You relate with others as usual.

¶146: A. I prefer being with my friends. And as for video games, well. If I have a room with plenty of space and I play being a robot, I do this and that, and it’s OK. But I think it’s all right if you play for half an hour or an hour sat down. I have not practiced moving my whole body in any video game…

¶147: B. But in your free time. I mean, this I did at school. But in my free time, I want to do what I feel like, and I´d actually prefer to keep still.

¶148: A. Me too.

¶149: D. Besides, I can watch TV.

¶150: C. For me, it depends on how I feel. If I´m tired or whatever, OK. Or if I feel normal, OK. But when, for example, I´m feeling “hey, I want to go outside” all the time and my parents won’t let me, then I use the video game.

¶151: A. When it’s raining and you can’t go out.

¶152: C. Yes of course, it depends on the situation.

¶153: D. And if you can talk with friends, so much the better.

<Files\\24 Grupo discusión alumnado 6º 2> - § 1 Coded references [Coverage 5,27%]

Reference 1 - Coverage 5,27%

¶20: D. I already knew Just Dance, so I didn’t catch my eye. But now after what you’ve done, I like it a lot and have bought it.

¶21: C. I´ve set it up. I mean I´ve used the website.

¶22: E. So have I. I thought that dancing and all that was great fun because you can meet your objectives. And if you can’t do what you’ve planned, you can sort it out. For example, the song, I mean the dance you can’t plan getting right, you can practice it several times until you master it.

<Files\\25 Grupo discusión alumnado 6º 3> - § 2 Coded references [Coverage 9,75%]

Reference 1 - Coverage 3,07%

¶4: A. Well I think this project is very good as it helps children to think who, apart from playing, many now play video games, although some children practice exercise while playing. That’s great. For example, Just Dance. It’s a video game that you can use to exercise with, which is a good idea.

Reference 2 - Coverage 6,68%

¶62: D. Yes, but if you wanted to lose a lot of weight, you’d be better running or walking. But if you want to lose just a couple of pounds, you can start dancing with the video game, for example.

¶63: B. I think it you don’t get tired the same. I think that if you dance every day, it’d be the same as if you run every day because you end up moving around. In fact I have Just Dance at home, which is downloaded in my mobile because I really like it. When I told my dad that I wanted to buy Just Dance, he was really pleased because he saw that your technique worked.

¶64: C. Yes. When I feel like doing exercise, I always dance. I have no video games, but if I did, I´d prefer to dance with no game.

**Category: 2.2.1. Advantages**

<Files\\15 Grupo discusión alumnado 6º grupo 01> - § 1 Coded references [Coverage 20,31%]

Reference 1 - Coverage 20,31%

¶34: D. I think that it was good because you could see everything you improved in, and green and yellow points told you that you’d improved and if you had done it well. I liked it because you could see everything you’d achieved in all the activities.

¶35: C. You could improve your scores.

¶36: B. I liked it because I could see what I was doing. That’s what I liked. At times, though, it stressed me a bit because we did it properly and wanted to improve. But when my phone stitched off, it wasn’t possible…

¶37: E. I dropped my phone.

¶38: B. I wasn’t bothered about a low score. But I got a bit stressed because I was sweating, tired and… “is that what I get?”. But it helped you to improve.

¶39: A. I liked it. Other activities without points would be better.

<Files\\23 Grupo discusión alumnado 6º 1> - § 2 Coded references [Coverage 7,65%]

Reference 2 - Coverage 6,33%

¶134: C. I quite liked it. I also thought it was good having an application which we can look at if we wanted to see our points instead of asking you, and all that. I didn’t see, but I thought that was quite good.

¶135: A. I tried, but couldn’t see it.

¶136: C. It helps you to no longer feel ashamed. I mean, if you wanted to try getting that point, you had to dance more and move around more. All this helps you to not feel ashamed.

¶137: D. Yes, you must make an effort to get a point and to know that you’re doing it properly. And you must continue that way. It helps you score more points.

¶138: A. When I scored a point, it was like “well done me”.

<Files\\24 Grupo discusión alumnado 6º 2> - § 1 Coded references [Coverage 14,59%]

Reference 1 - Coverage 14,59%

¶97: A. I thought it was quite good because, for example, if we cooperated, the group would score more points. So it’s a way when some people, say, don’t want to cooperate, we’d say “right, let’s cooperate to score a point”.

¶98: B. I think the points system is cool but, for me, points should also be scored for attitude. Because, perhaps, some find dancing harder, but show the right attitude to dance better, you know what I mean? The same goes for the Class Dojo points, you should add points for attitude.

¶99: A. Yes, and also for attention too because in that way…

¶100: C. I thought it was good for improving or for seeing if you had done it properly. For example, if you didn’t get a point, you think about what you can do to improve. If you got a point, you’d feel proud about yourself, and continue.

¶101: D. I think it’s a fair scoring system because you deserve one point if you do things well. And if you don’t make an effort, you aren’t given a point. You have to make an effort and do well to be given a point. It’s fair because you get a point if you deserve it.

¶102: E. I like it because when I got a point, I felt more motivated to do things, and I continued making an effort.

¶103: A. I thought it was good because for, example, some people wanted to be first, and if you said that, you’d make more effort. Whether you managed it or not, you’d make more effort.

¶104: B. Yes, you feel more motivated to be successful at it. If you’re not first you think «Well, I can manage it another week”.

<Files\\26 Grupo discusión alumnado 6º 4> - § 2 Coded references [Coverage 31,35%]

Reference 1 - Coverage 27,57%

¶27: C. Well for example, if you did worse in the individuals, and your classmates helped you in the group, you could get more points from being in the group.

¶28: D. Yes, the points helped.

¶29: A. Yes because, for example, if you set an easy goal, then you will dance better and make up on points.

¶30: B. It depends on the purpose. It depends on the goal you set. If you set, for example, “improve more” by the end of the month, then that’s it. But if you set, I don’t know, “be the best” …

¶31: A. The part where you can repeat the song is good because you have chances to improve.

¶32: C. Yes, you had chances to improve your score. For example, you got three starts the first time, but then you could try for four.

¶33: D. That’s right because, as you already know the dance from before, you have learnt some steps.

¶34: C. You remembered some dance steps, so you did it better.

¶35: D. That’s right, and some steps were repeated quite a lot, so you got the hang of the dance in the end. Then when I’d mastered it, I thought, “ah, fine, it’s my turn now to do this”.

¶36: C. I thought the classification was good because if I am too low, I can move up, and if I´m in a good position, I can stay where I am …

¶37: B. To keep fit.

¶38: C. To keep fit, yes.

¶39: A. It made me happy too because you came first anyway, which motivated you, you know?

Reference 2 - Coverage 3,78%

¶59: D. It didn’t matter if you forgot. But with Just Dance Now, as you get points, you lose points if you forget a part or stop moving your hand.
