# Supplementary material for: Analysis of the applicability and utility of a gamified didactics with exergames at primary schools: Qualitative findings from a natural experiment
Source: PLoS One. 2020 Apr 10;15(4):e0231269. doi: 10.1371/journal.pone.0231269 (PMC7147727; doi:10.1371/journal.pone.0231269)
Supplement: S1 Checklist — (DOCX) [file pone.0231269.s003.docx]

S3. Checklist. Consolidated criteria for reporting qualitative studies (COREQ): 32-item checklist

This is the COREQ checklist which accompanies the qualitative data gathering analysis for this dataset.

Authors are as follows:

Alejandro Quintas-Hijós^1^

Carlos Peñarrubia-Lozano^2^

Juan Carlos Bustamante^3^

1. Department of Education Sciences, University of Zaragoza, Huesca, Spain,
2. Department of Musical, Plastic and Corporal Expresion, University of Zaragoza, Zaragoza, Spain.
3. Department of Psychology and Sociology, University of Zaragoza, Zaragoza, Spain.

| **No** | **Item** | **Guide questions/description** |
| --- | --- | --- |
| **Domain 1: Research team and reflexivity** |  |  |
| Personal Characteristics |  |  |
| 1. | Interviewer/facilitator | Which author/s conducted the interview or focus group?  First author and second author. The third author was not involved in data collection. |
| 2. | Credentials | What were the researcher's credentials? *E.g. PhD, MD*  The first author is a PhD candidate. The second and third authors are PhD. |
| 3. | Occupation | What was their occupation at the time of the study?  First author: Assistant Professor, second author: PhD Assistant Professor, third author: Associate Professor |
| 4. | Gender | Was the researcher male or female?  All the authors are male |
| 5. | Experience and training | What experience or training did the researcher have?  The authors have 7, 10, and 13 years of experience as researchers. The first author is an educational scientists who specialises in human-computer interaction for children. The second author is a PE researcher. The third author is a neuropychological scientist and methodologist. |
| Relationship with participants |  |  |
| 6. | Relationship established | Was a relationship established prior to study commencement?  Children met the first author for the first time when the study began as he was the teacher of the interventions. Children met the second author at the time of the interviews; that is, at the end of the interventions. |
| 7. | Participant knowledge of the interviewer | What did the participants know about the researcher? e*.g. personal goals, reasons for doing the research*  The participants knew that the first and second researchers were from a local university. The informed consent forms, which the participants’ parents signed, explained the general study purposes. |
| 8. | Interviewer characteristics | What characteristics were reported about the interviewer/facilitator?  Children knew that the first author would be a teacher invited for 1 month. They were told that we needed their help to improve education and that we appreciated the comments they would make at the end of interventions. |
| **Domain 2: study design** |  |  |
| Theoretical framework |  |  |
| 9. | Methodological orientation and Theory | What methodological orientation was stated to underpin the study?  The thematic analysis focused on identifying themes relating to behavior. Content analysis was chosen as methodological orientation. |
| Participant selection |  |  |
| 10. | Sampling | How were participants selected?  Of the nine schools invited to participate in it, only one declined because it considered that the intervention incompatible with the curriculum. Finally, the researchers chose four schools according to the following criteria: the adequacy of their material (Wi-Fi connectivity, facilities), the diversity of the public/private school’s, schools from different cities (Huesca and Zaragoza, Spain), students’ ethnic and socio-economic diversity, teaching staff’s positive predisposition and accessibility for researchers. |
| 11. | Method of approach | How were participants approached? e*.g. face-to-face, telephone, mail, email*  Children and teachers were contacted face-to-face in the last classes of interventions to arrange interviews. |
| 12. | Sample size | How many participants were in the study?  Eight teachers and 417 students took part in the study. |
| 13. | Non-participation | How many people refused to participate or dropped out? Reasons?  One student did not participate in the study because his parents did not provide consent as he had Asperger’s and they did not want his data to be collected. The student participated in the intervention, because they were normal classes, but no data were taken.  The students of a special school did not complete the OQQ due to its generalized disruptive behavior and lack of literacy competence. |
| Setting |  |  |
| 14. | Setting of data collection | Where was the data collected? e*.g. home, clinic, workplace*  Children’s interviews and focus groups were conducted in the different rooms available at schools during the school period. Teacher interviews also took place in classrooms during breaks. The OQQ was applied during the last 15 minutes of the intervention. |
| 15. | Presence of non-participants | Was anyone else present besides the participants and researchers?  There was no-one else present other than participants and researchers |
| 16. | Description of sample | What are the important characteristics of the sample? *e.g. demographic data, date*  The qualitative data herein reported were collected during the natural experiment with a nonrandomised control conducted between October 2017 and June 2018 in four primary schools of Aragon (Spain). The student sample comprised 417 students (53.2% girls, n=222; 46.8%, n=195) from four primary schoo. Their mean age was 11.1 (SD=1.7), and 50.4% of the sample studied Year 6 (aged 10-11 years; n=210) and 49.6% studied Year 7 (11-12 years; n=207) in Primary Education schools. |
| Data collection |  |  |
| 17. | Interview guide | Were questions, prompts, guides provided by the authors? Was it pilot tested?  The authors prepared a set of questions for the semi-structured interviews and focus groups in advance. It was not pilot-tested. |
| 18. | Repeat interviews | Were repeat interviews carried out? If yes, how many?  There were no repeat interviews |
| 19. | Audio/visual recording | Did the research use audio or visual recording to collect the data?  All the interviews and focus groups were audio-recorded. Interviewers contents were written notes by hand. All the interviews and discussion groups were audio-recorded and video-recorded to be able to differentiate voices later. |
| 20. | Field notes | Were field notes made during and/or after the interview or focus group?  The firstauthor wrote the field notes after the intervention sessions, but not after the interviews. |
| 21. | Duration | What was the duration of the interviews or focus group?  The OQQ took 15 minutes to complete on overage. Focus group interviews took 20 minutes. Individual semi-structured interviews took 25 minutes on average |
| 22. | Data saturation | Was data saturation discussed?  No, it was not. |
| 23. | Transcripts returned | Were transcripts returned to participants for comment and/or correction?  No. Infographic panels were designed for each school by summarizing the findings during the interventions in didactic terms. |
| **Domain 3: analysis and findings** |  |  |
| Data analysis |  |  |
| 24. | Number of data coders | How many data coders coded the data?  The second author was the only data coder. The first author checked the coding. |
| 25. | Description of the coding tree | Did authors provide a description of the coding tree?  Yes, in the Appendix |
| 26. | Derivation of themes | Were themes identified in advance or derived from the data?  The analysis was both deductive (to study previously raised problems and elements) and inductive (allowed new issues to emerge). The original classification tree was built based on the previously considered concept.  The final thematic analysis was promoted by previously established themes that were, mixed with the emerging ones seen in the data. |
| 27. | Software | What software, if applicable, was used to manage the data?  The Nvivo software (version 11, <https://www.qsrinternational.com/nvivo/home>)  was used to analyze all the contents from interviews and focus groups.  Spss (version 21.0, <https://www.ibm.com/es-es/analytics/spss-statistics-software>) for the OQQ. |
| 28. | Participant checking | Did participants provide feedback on the findings?  This was partially done. Teachers were informed of the study results. Students only received any relevant educational qualifications and assessments. |
| Reporting |  |  |
| 29. | Quotations presented | Were participant quotations presented to illustrate the themes / findings? Was each quotation identified? e*.g. participant number*  Yes, a real quote from a participant was used in each thesis on all the themes. An in-depth hermeneutical analysis was performed.  Yes, each quotation was identified. The coding used to identify each extract was based on four digits: the first number refers to the specific interview or discussion group (numbered from 1 to 26); the first letter indicates gende ("b" for boy, "g" for girl); the second number indicates the paragraph number in each transcribed document; the second letter indicates the category of participants ("s" for student, "t" for teacher). |
| 30. | Data and findings consistent | Was there consistency between the data presented and the findings?  Yes, an attempt was made to gain consistency through various triangulations: data triangulation of the both control and experimental intervention results, data triangulation of both teachers’ and pupils’ perspectives by considering their expectations and views of interventions; data triangulation of four different qualitative data collection techniques; quantitative and qualitative data triangulation. |
| 31. | Clarity of major themes | Were major themes clearly presented in the findings?  The major themes are clearly identified by section headings (“applicability”, “utility” and “differentiation between the two”) |
| 32. | Clarity of minor themes | Is there a description of diverse cases or discussion of minor themes?  Diverse cases were considered, for example negative cases or unexpected examples as set out by previous literature. Minor themes are mentioned, such as “shame”. |
